# Chemical Composition, Nutritional Value, and Biological Evaluation of Tunisian Okra Pods (*Abelmoschus esculentus* L. Moench)

**DOI:** 10.3390/molecules25204739

**Published:** 2020-10-15

**Authors:** Mariem Haj Romdhane, Hassiba Chahdoura, Lillian Barros, Maria Inês Dias, Rúbia Carvalho Gomes Corrêa, Patricia Morales, Maria Ciudad-Mulero, Guido Flamini, Hatem Majdoub, Isabel C. F. R. Ferreira

**Affiliations:** 1 Laboratory of Interfaces and Advanced Materials, Faculty of Sciences of Monastir, University of Monastir, Monastir 5000, Tunisia; mariemhajromdhane.lima@gmail.com (M.H.R.); hatemmajdoub.fsm@gmail.com (H.M.); 2 Laboratory of Bioresources: Integrative Biology and Exploiting, Higher Institute of Biotechnology of Monastir, University of Monastir, Monastir 5000, Tunisia; hassiba_chahdoura@yahoo.fr; 3 Centro de Investigação de Montanha (CIMO), Instituto Politécnico de Bragança, Campus de Santa Apolónia, 5300-253 Bragança, Portugal; rubia.correa@unicesumar.edu.br (R.C.G.C.); iferreira@ipb.pt (I.C.F.R.F.); 4 Program of Master in Clean Technologies, Cesumar Institute of Science Technology and Innovation (ICETI), Cesumar University-UniCesumar, Parana 87050-390, Brazil; 5 Departament of Nutrition and Food Science, Faculty of Pharmacy, Complutense University of Madrid, Plaza Ramón y Cajal, s/n, E-28040 Madrid, Spain; patricia.morales@farm.ucm.es (P.M.); mariaciudad@ucm.es (M.C.-M.); 6 Dipartimento di Farmacia, via Bonanno 6, Università di Pisa, 56126 Pisa, Italy; guido.flamini@unipi.it; 7 Centro Interdipartimentale di Ricerca “Nutraceutica e Alimentazione per la Salute” Nutra Food, Università di Pisa, 56124 Pisa, Italy

**Keywords:** okra pods, proximate composition, volatile compounds, phenolic compounds, cytotoxicity, enzyme inhibitory potential, analgesic activity

## Abstract

The aim of this work was to perform an unprecedented in-depth study on the bioactive phytochemicals of *Abelmoschus esculentus* L. Moench Tunisian landrace (Marsaouia). For this purpose, its nutritional, aroma volatile, and phenolic profiles were characterized, and sundry biological activities were assessed in vitro. The approximate composition revealed that total dietary fiber as the most abundant macronutrient, mainly insoluble dietary fiber, followed by total carbohydrates and proteins. In addition, okra pods were rich in K, Ca, Mg, organic acids, tocopherols, and chlorophylls. Gas Chromatography-Electron Impact Mass Spectrometry (GC-EIMS) analysis showed that oxygenated monoterpenes, sesquiterpene hydrocarbons, and phenylpropanoids were the predominant essential volatile components in *A. esculentus* pods. A total of eight flavonols were detected by High-Performance Liquid Chromatography coupled to a DAD detector and mass spectrometry by electrospray ionization (HPLC-DAD-MS/ESI); with quercetin-3-*O*-glucoside being the majority phenolic component, followed by quercetin-*O*-pentosyl-hexoside and quercetin-dihexoside. This pioneering study, evidences that Tunisian okra display promising antioxidant and cytotoxic actions, in addition to relevant inhibitory effects against α-amylase and α-glucosidase enzymes, and interesting analgesic activity.

## 1. Introduction

Food and nutrition are closely associated with human health. Through the past few decades, science has made great efforts to unravel how nutrients and functional ingredients modulate human physiology and responses. The consumers’ growing interest in the role of diet in achieving well-being is a reflection of some discoveries already made. For instance, the high intake of plant products was associated with a reduced risk of chronic diseases, such as atherosclerosis, cancer, and diabetes [1]. Consequently, several conventional and non-conventional vegetables have been critically investigated for their nutritional contents [2]. Among them, there is okra, *Abelmoschus esculentus* (L.) Moench, an annual herb belonging to the Malvaceae family. This economically important vegetable crop, native to Africa, is now widely grown in Southern Europe, Middle East, Asia, and America [3].

Since ancient times, infusions and decoctions of *A. esculentus* fruits (pods) were employed in folk medicine as a diuretic and for treating diarrhea, acute inflammation, stomach, and bowels irritation, catarrhal infections, gonorrhea and dysuria, dental ailments, bronchitis, and pneumonia [4]. The whole okra plant is edible; however, the immature pods has a unique taste and are traditionally used (whether fresh, dried, boiled, or fried) in the preparation of soups, salads, and stews [5].

The excellent content of phenolics and pectins of *A. esculentus* fruits, greatly dependent of their seed fraction, is well-known [6,7]. Okra pods are mucilaginous, low in calories, but nutritionally rich and a good source of edible dietary fibers. Studies have shown that they contain important bioactive compounds such as vitamin C, carotene, thiamine, folic acid, riboflavin, oxalic acid, niacin, and amino acids. Furthermore, pods are good sources of minerals (K, Ca, P, and Mg) and very low in cholesterol and saturated fats [4]. In addition to its direct consumption, okra fruits have also been exploited by the pharmaceutical and food industries as emulsifier additives, blood volume expanders, and in drug tablet formulation, due to their high content in biopolymers and bioactive molecules like polysaccharides and flavonoids [5].

In the past five years, sundry in-vitro and in-vivo research evidenced that *A. esculentus* extracts display prominent biological activities such as cardio, renal, gastro, and neuro-protective effects [4], as well as antioxidant, antidiabetic and antihyperlipidemic [3], antifatigue, antibacterial [6], anti-inflammatory, and analgesic [8] properties. Nevertheless, and despite the aforementioned reports, to the best of the authors’ knowledge, an advanced characterization of its phenolic and volatile constitution is lacking.

Based on the above considerations, the aim of the present study was to perform an unprecedented in-depth study on the phytochemical profile and bioactivities of *A. esculentus* Tunisian landrace (Marsaouia), investigating its use in a daily-basis diet and also as a source of high-added value molecules with bioactive capacity to be exploited by the pharmaceutical and food industries. For that manner, the nutritional composition of this material was estimated and characterized in terms of phenolic and volatile compositions, besides antioxidant and cytotoxic potentials. The inhibitory potential against α-amylase and α-glucosidase was also assessed. Finally, analgesic activity using acetic acid induction was investigated.

## 2. Results and Discussion

### 2.1. Nutritional Features

Nutritional value and sugar composition of Tunisian okra pods are presented in Table 1. Okra is more a diet food than staple, and due to the fact that there is no comparative literature with the okra pods, the discussion of results will be carried out in comparison to other species of plants that serve the same purpose, such as quinoa and amaranthus, that are now also being used as alternative sources of nutritional compounds. The fruits have high water content (81.9%) and low fat content (0.066 g 100 g^−1^ fresh weight-fw). Dietary fibers were the most abundant macronutrients (8.16 g 100 g^−1^ fw), followed by carbohydrates (4.86 g 100 g^−1^ fw) and proteins (3.55 g 100 g^−1^ fw), which contribute to the calorific value of okra pods (50.57 Kcal 100 g^−1^). Furthermore, proteins content is considered high compared to other okra cultivars: Clemson spineless and dwarf long green varieties [9]. Okra pods present lower energetic intake (lower fat and carbohydrates) and lower protein intake when compared to quinoa from Brazil [10] and Chile [11], but a very similar profile in terms of water, fiber, protein, and fat content with various species of Amaranthus plants [12]. Total dietary fiber content of the analyzed Tunisian okra pods was 8.16 g 100 g^−1^, fw. As dietary fiber intake is associated with several health benefits, the European Food Safety Authority recommended a dietary fiber intake of 25 g per day in adults [13]. The consumption of 100 g of okra could cover a 32.6% of the official recommendation. Okra contains both insoluble (4.73 g 100 g^−1^ fw) and soluble (3.43 g 100 g^−1^ fw) dietary fiber. Each of these fractions has important properties related to human health. In the case of insoluble dietary fiber, it contributes to the normal function of the intestinal tract and it also has an important role in the prevention of gastrointestinal diseases such as colonic diverticulosis. Soluble dietary fiber is fermented by gut microbiota and regulates the metabolism of lipids, showing hypocholesterolemic properties. It is known that okra is beneficial in the control of hyperlipidemia and other associated metabolic disorders, as okra dietary fiber is responsible for hypolipidemic activity [14]. It is also important to state that okra pods also present higher amounts of crude fiber than other plants like quinoa and amaranthus [11,12], plants that have also been increasingly used in a daily-basis diet for the substitution of other nutritional sources. 

Mineral composition of Tunisian okra pods is shown in Table 1. Potassium (337.67 mg 100 g^−1^ fw) was the main mineral found in the analyzed samples followed by calcium (266.62 mg 100 g^−1^ fw). This tendency and similar values were previously reported by other authors in different okra genotypes [6]. Among microelements, zinc (2.00 mg 100 g^−1^ fw) and iron (1.60 mg 100 g^−1^ fw) were found in higher amounts than copper (0.49 mg 100 g^−1^ fw) and manganese (0.13 mg 100 g^−1^ fw). Both macroelements and microelements are considered to be essential in human nutrition, with okra being an interesting food due to their mineral composition [4].

Regarding soluble sugars composition (Table 2), the main detected sugars were sucrose (110.40 g 100 g^−1^ fw), followed by fructose, glucose (34.8, 30.9 100 g^−1^ fw, respectively). However, trehalose was detected in a low amount (15.46 g 100 g^−1^ fw). Studies on the small and large commercial Greek cultivars “Pylaea” showed that the total sugar content is low compared to that found in the “Marsaouia” Tunisian variety (2.26 and 3.11 g 100 g^−1^ dw for the small and large “Pylaea”, respectively, against 3.46 g 100 g^−1^ dw for the “Marsaouia”) [6]. Moreover, a difference in carbohydrate constituents of “Marsaouia” and “Pylaea” was noticed. The presence of trehalose was detected only in the Tunisian variety, which could be explained by the fact that the harvest conditions, the nature of the soil, the climate, and the genotype could efficiently affect the carbohydrate constituents of okra.

Organic acid content of Tunisian okra pods is presented in Table 2. The major organic acid was citric acid, which constituted 54.64 g/100 g fw of total organic acid, followed by oxalic and malic acids, which presented 23.24 and 20.92 g/100 g fw respectively, of total organic acid. While fumaric acid and shikimic acids were detected in a lower amount. Oxalic acid and malic acid were the only organic acids detected in Greek genotypes [6]. Also, okra pods contain five times more organic acids than quinoa seeds from Brazil (4.6 g 100 g^−1^ fw) [10]. The composition in tocopherols, carotenoids, and chlorophyll are also shown in Table 2. γ-Tocopherol was the major tocopherol vitamer found, succeeded by α-tocopherol, while no other vitamers were identified. Chlorophylls and carotenoids are common organic food components giving a specific coloration to plants, since they are naturally present in it. Tunisian okra showed high β-carotene and lycopene content comparing to other okra genotypes such as Lashithi, Pylaea, and Veloudo. Chlorophyll, although not principally significant nutritionally, offered a measure of green vegetable color, an estimation of senescence for consumers, and significant effects on oxidation, wound healing, and inflammation [15]. Okra pods contain 3.53 mg/100 g fw of chlorophyll a and 2.43 mg/100 g fw of chlorophyll b. These results are similar to those found by [6].

### 2.2. Volatile Compounds Determination

The volatile compounds composition of Tunisian okra pods is presented in Table 3. A total of 35 volatile components were detected. The detected compounds accounted for 96.50% of the total aroma. Usually, each volatile component is distinguished by an odor threshold (varying from ng/mL to µg/mL), so even if among different samples, the qualitative composition is very alike, the aroma may vary when the relative proportions are dissimilar [16]. Quantitatively, the volatile profile displayed that non-terpene derivatives (38%), which are mainly straight-chain alkanes, alcohols as well as ketones and aldehydes, and oxygenated monoterpenes (22.20%) (phenols, alcohols, ethers, and ketones) were found to be the main classes of volatiles in okra pods. Furthermore, sesquiterpene hydrocarbons, phenylpropanoids, and monoterpene hydrocarbons were moderately present with 11.40, 11.10, and 9.50% respectively. Apocarotenes were slightly represented (4.30%).

The main volatile constituents of okra pods were (E)-anethole (6.90%), limonene (6.70%), β-caryophyllene (5.40%), decanal (4.60%), and carvone (4.50%) (Table 3). Anethole is known for its cytotoxic, mutagenic, anti-inflammatory, and analgesic activities, as well as its antimicrobial, antifungal and fumigant activities [17]. Limonene, which was present at 6.70 % of the total volatile compounds of okra pods, exhibits cytotoxic and antibacterial activities [18]. Furthermore, several biological activities are referred to β-caryophyllene, such as antibiotic, antioxidant, anti-inflammatory, anticarcinogenic, as well as local anesthetic effects [19]. Molfetta [16] found a different volatile profile than that presented herein when assessing seed samples of Italian *A. esculentus* for their volatile emission using solid phase micro-extraction. Among the 17 compounds detected by the authors, isopentyl 2-methyl butanoate (24.5–59.1%) and heptanoic acid 2-methylbutyl ester (6.6–13.5%) were the predominant ones. That said, to the best of the authors’ knowledge, the present report is the first on the volatile composition of fresh okra pods.

### 2.3. Phenolic Profile

The peak characteristics (retention time, wavelength of maximum absorption, and mass spectral data), tentative identification, and quantification of phenolic compounds found in *Abelmoschus esculentus* are presented in Table 4. A total of eight compounds were identified as being all assigned as flavonol glycoside derivatives. Peaks 1, 2, 3, 4, and 5 were all identified as quercetin glycosides based on their UV spectra (λ_max_ around 353 nm) and the production of MS^2^ fragment ion at *m*/*z* 301. Likewise, peaks 6, 7, and 8 were identified as kaempferol derivatives by the registered fragment ion at *m*/*z* 285. Quercetin-3-*O*-glucoside was the predominant extracts’ constituent accounting for almost 32% of the phenolic content (1.50 ± 0.01 mg. g^−1^), followed by quercetin-*O*-pentosyl-hexoside (0.90 ± 0.02 mg. g^−1^) and quercetin-dihexoside (0.79 ± 0.01 mg.g^−1^) (Table 4). To the best of the authors’ knowledge, this was the first report on the identification of such compounds in *A. esculentus* and only the second work in the literature on okra pods regarding phenolic characterization.

Recently, Meinhart et al. [20] assessed the composition in chlorogenic acids and caffeic acid of three Brazilian okra fruit samples via high-performance liquid chromatography, therefore describing distinct profiles than those obtained in the present work. Authors detected the following compounds (medium values): 3-*O*-caffeoylquinic (25.5 mg.k^−1^ fw), 5-*O*-caffeoylquinic (1.6 mg.k^−1^ fw), and 3,5-*O*-dicaffeoylquinic (1.25 mg^−1^ fw). Regarding the total phenolic content (TPC), *A. esculentus* pods extract presented a concentration of 4.75 mg. g^−1^ (Table 4), which unfortunately cannot be compared with other authors. Only Xu et al. [7] investigated the phenolic fractions of several flours obtained from okra seeds and seedless pods; however, the values of TPC were reported in gallic acid equivalents, between 288.2 and 3426.2 mg GAE/100 g. It is known that other groups of molecules (e.g., reducing sugars) found in important amounts in vegetable extracts can also reduce the Folin Ciocalteu reagent, consequently producing overestimated values of TPC via this method [21].

It is well-established that a major part of the quercetin and kaempferol found in plant matrices is attached to sugar moieties rather than in the free form. A broad spectrum of bioactivities was documented for these flavonoid glycosides, including antimicrobial, antioxidant, and antiproliferative effects [22].

### 2.4. Bioactive Proprieties

#### 2.4.1. Antioxidant Potential

DPPH (2,2-diphenyl-1-picrylhydrazyl) is a relatively stable free radical; antioxidants are used to reduce free radicals by providing hydrogen atoms or electrons. Therefore, the DPPH radical can be used to evaluate antioxidant free radical scavenging activity. The FRAP (ferric reducing antioxidant power) assay takes advantage of electron-transfer reaction. The transfer of the electron from the antioxidant to the probe resembles the redox titration in classical chemical analysis, because there is not a competitive reaction involved and there is no oxygen radical in the assay [21]. β-carotene bleaching assay is also a common way to evaluate the antioxidant potential of hydroxylated fullerenols. As shown in Table 5, okra displayed outstanding antioxidant activities on DPPH. (EC_50_ = 1.03 mg/mL), FRAP (EC_50_ = 0.89 mg/mL), and β-carotene bleaching (EC_50_ = 0.47 mg/mL). The high antioxidant activities may be explained by the high content of phenolic compounds holding hydroxyl groups, which donates protons to free radicals to scavenge them.

#### 2.4.2. Cytotoxic Activity

Considering cancer now causes more deaths than all coronary heart diseases or all strokes [23], the cytotoxic potential of Tunisian okra pods against human non-small cell lung cancer (NCL-H460), human breast adenocarcinoma cell line (MCF-7), human cervical cancer cells (HELA), and human liver hepatocellular cells (HEPG-2) were investigated. The results showed that okra pods induced cytotoxicity in a dose-dependent manner and inhibited the development of NCL-H460, MCF-7, HELA, and HEPG-2 cells to 50% at concentrations of 49.62, 56.40, 67.27, and 167.95 mg/mL, respectively. Taking into account okra pods composition, its cytotoxicity could be assigned to its terpenoid compounds, which permeabilize and disrupt cell membranes, particularly mitochondrial, leading to reactive oxygen species release. It was reported that β-caryophyllene, which constitute 5.4% of total volatile compounds, promotes the passage of paclitaxel via cancer cells membrane and therefore enhances its anticancer potential. Limonene, which represented 6.70% of total volatile compounds, improved oxidative stress, along with docetaxel, in cancer cells, which was followed by a decreased level of glutathione and activation of apoptosis and caspases [18]. Besides, Eugenol, which constituted 4.20% of total volatile compounds, induced apoptosis of cervical cancer cells without toxicity to healthy cells and potentiated the effect of chemotherapeutic agents [24]. On the other hand, the cytotoxicity of Tunisian okra pods could be attributed to its phenolic compounds. Polyphenols are recognized as natural antioxidants; however, their high concentrations exhibit pro-oxidant properties and they could therefore incite cytotoxicity [1].

#### 2.4.3. α-Amylase and α-Glucosidase Inhibitory Activities

Diabetes mellitus (DM) impacts around 5% of the human population and a therapy without any secondary effects is still unknown to scientists. Type 2 DM is a heterogeneous illness resulting from the dynamic interrelationship between defects in insulin action and insulin secretion. Such a deficiency leads to increased blood glucose levels, which harm the body’s systems, particularly the blood vessels. Studies of the natural process of the disease have shown that it is characterized by a progressive prostration of cells. People affected by DM type 2 are insulin-resistant and usually have a metabolic syndrome, a multifactorial intervention including aggressive treatment of dyslipidaemia and arterial hypertension [25]. One of the therapeutic approaches for treating type 2 DM is to reduce the post-prandial glucose levels. In fact, this could be realized by delaying the absorption of glucose via the inhibition of the carbohydrate-hydrolysing enzymes, α-amylase, and α-glucosidase, present in the brush border of the small intestinal, which are responsible for the degradation of oligosaccharides and disaccharides to monosaccharides adequate for absorption [26]. The potential role of polysaccharides extracted from *Abelmoschus esculentus*, have been investigated by a few authors [3,22]. Therefore, the study of the hypoglycemic potential of the whole vegetable seemed interesting. In the present study, Tunisian okra pods showed a relevant inhibitory potential on α-amylase (IC_50_ = 125 µg/mL) and α-glucosidase (IC_50_ = 110 µg/mL), as shown in Table 5. These findings lead to the conclusion that the high inhibitory potential of Tunisian *Abelmoschus esculentus* could be explained by the synergic effect of polysaccharides and flavonoids present in okra fruit, and is not only due to the polysaccharides group from okra, as previously reported by Yuang et al. [22]. In fact, flavonoids are reported to be effective in the inhibition of the α-amylase and α-glucosidase activities, via the inhibition of glucose transporters. A recent study had shown that the capacity of inhibition is related to the number of hydroxyl groups on the B ring of the flavonoid backbone. The interaction is established by the formation of hydrogen bonding between hydroxyl groups of the ring A (in position R6 or R7) and the ring B (in position R4′ or R5′) of the polyphenol ligands and the catalytic residues of the attachment site, and formation of a conjugated π-system that maintains the interaction with the active site [26].

#### 2.4.4. Analgesic Activity

The inhibition percentages of writhing for Tunisian *Abelmoschus esculentus* at different concentrations are presented in Table 5. The reference drug inhibited 63.65% of the writhing response induced by acetic acid. The results showed that okra pod extract induces in a dose-dependent manner the antinociceptive potential. Okra pods extract reduced significantly the number of writhing, which is related to the release of endogenous substances including histamines, serotonin, bradykinin, and prostaglandin [27]. The antinociceptive activity of the extract of Tunisian okra pods at 100 mg/kg (58.11%) were found to be higher than that of the methanolic extract of okra roots at the same concentration (36.99%) [28] and okra water extract (30.76%) [29]. More recently, Alves et al. [8] evidenced the antinociceptive and anti-inflammatory effects of a lectin extracted from Brazilian okra’s seeds in the formalin-induced temporomandibular joint inflammatory hypernociception in rats. 

The pain stimulus induced by the acetic acid leads to the liberation of free arachidonic acid from the tissue phospholipids. Studies suggest that the response is mediated by peritoneal mast cells acid sensing ion channels and the prostaglandin pathways [30]. It is well known that analgesic drugs alleviate the inflammatory pain by reducing the creation of pain mediators at the peripheral target sites where prostaglandins and bradykinins are suggested to play an important role in the pain mechanism and flavonoids inhibiting the writhing will have anti-nociceptive effect essentially by the inhibition of prostaglandin synthesis, a peripheral process of pain prevention [31]. Therefore, the peripheral anti-nociceptive action of Tunisian okra extract was likely exerted by interfering with the local reaction caused by the irritant or by inhibiting the synthesis, release, and/or antagonizing the action of pain mediators at the target sites. Interestingly, bioactive components like flavonoids and steroids, besides triterpenes in part, have been shown to possess analgesic activity [30,31].

## 3. Materials and Methods

### 3.1. Plant Material

Local landrace okra pods “Marsaouia” (*Abelmoschus esculentus* L. Moench) were purchased from a local market (Bou Argoub, Tunisia). Fresh pods were rinsed with distilled water and frozen, for posteriorly lyophilization. Then, the lyophilized material was milled (Moulinex mixer, Model DD306141) until the obtaining of a homogenous fine dried powder, stored at 25 °C, and protected from light for further analysis.

### 3.2. Chemical Composition

#### 3.2.1. Nutritional Value

Okra pods were analyzed in terms of macronutrients (moisture, proteins, fat, carbohydrates, and ash), according to the AOAC (Association of Official Analytical Chemists) procedures [32]. Crude protein content (N × 6.25) was estimated using the macro-Kjeldahl method (AOAC 978.04); dietary fiber (soluble, insoluble and total) was quantified using enzymatic-gravimetric methods (AOAC 993.19 and 991.42); soxhlet extraction with petroleum ether was used to estimate the crude fat content (AOAC 920.85); total carbohydrate content was obtained by difference, and finally the energetic value was calculated through the formula: energy (kcal/100 g fw) = 4 × (g protein + g available carbohydrate) + 2 × (g dietary fiber) + 9 × (g fat).

Total mineral content (ashes) and mineral element analysis were performed by dry ash mineralization at 550 °C (AOAC 923.03). Minerals were extracted in an acid mixture (1 mL HCl 0.5 mL/mL + 1 mL HNO_3_ 0.5 mL/mL) and made up to 50 mL of distilled water. Different dilutions were prepared in order to quantify microelements (Fe, Cu, Zn, and Mn, >99% purity, Merck, Darmstadt, Germany) and macroelements (Ca, Mg, Na, and K, >99% purity, Merck, Darmstadt, Germany) by atomic absorption spectroscopy (AAS) with air/acetylene flame in a Perkin-Elmer 2280 spectrophotometer [33]. For Ca and Mg determination, a dilution with La_2_O_3_ (58.6 mg/L deionized water:HCl) was performed in order to avoid interferences. For Na, K, Ca, Mg, Cu, Fe, Mn, and Zn analysis, the used wavelengths were 589.0, 766.5, 422.7, 285.2, 324.8, 248.3, 279.5, and 213.8 nm, respectively. Slit was 0.7 nm except for K, Fe, and Mn, in which 0.2 nm was applied.

#### 3.2.2. Volatile Compound Analyses

Solid phase Micro-extraction (SPME) equipment (Supelco, Sigma-Aldrich, Bellefonte, PA, USA) covered with poly-dimethylsiloxane (PDMS, 100 μm) were used to sample the headspace of a dry flower inserted into a 5-mL vial and let to equilibrate for 30 min. SPME sampling was performed via the same new fiber, circumstanced as claimed by the manufacturer’s instructions, for all the analyses. To assure temperature stability, Sampling was realized in an air-conditioned room (22 ± 1 °C). After the equilibration time, the fiber was exposed over 50 min to the headspace at room temperature. Once sampling was realized, the fiber was removed and switched to the injection port of the Gas Chromatography-Mass Spectrometry (GC-MS) system. Blanks were carried out before SPME extraction. GC-electron impact mass spectrometry (EIMS) analyses were performed using a Varian (Varian inc., Palo Alto, CA, USA) CP3800 gas chromatograph with a DB-5 capillary column (30 m × 0.25 mm × 0.25 μm; Agilent, Santa Clara, CA, USA) and a Varian Saturn 2000 ion trap mass detector. The used analytical conditions were: injector temperature of 220 °C, transfer line temperature of 240 °C, oven temperature programmed from 60 to 240 °C at 3 °C min^−1^, helium as carrier gas at 1 mL min^−1^, splitless injection. Volatile identification was fulfilled by comparing their retention times, MS spectra, and linear retention indices (LRI), along with computer matching with commercial and in-house library mass spectra and data [34,35].

#### 3.2.3. Soluble Sugars

Soluble sugars were assessed via HPLC coupled to an RI detector (Knauer, Smartline system 1000, Berlin, Germany) applying the internal standard (IS, melezitose, Sigma-Aldrich, St. Louis, MO, USA) method, as formerly described by the authors [36]. Mobile phase was composed by an acetonitrile: water mixture (70:30 *v*/*v*, acetonitrile HPLC-grade, Lab-Scan, Lisbon, Portugal), whereas separation was completed using a Eurospher 100-5 NH2 column (4.6 × 250 mm, 5 µm, Knauer). The results were recorded and treated using Clarity 2.4 software (DataApex, Prague, Czech Republic).

#### 3.2.4. Organic Acids

The dehydrated and powdered fruits and stems were investigated for their organic acid composition following the protocol established by the group [37]; an ultra-fast liquid chromatography coupled to photodiode array detector (UFLC-PDA; Shimadzu Coperation, Kyoto, Japan) was used. The separation of the compounds was performed in a SphereClone (Phenomenex) reverse phase C18 column (5 μm, 250 × 4.6 mm i.d) thermostated at 35 °C, using 3.6 mM sulphuric acid solution as eluent in a flow rate of 0.8 mL/min. The quantification was achieved by comparison of the peak area recorded at 215 nm as the preferred wavelength. For quantitative analysis, a calibration curve with known concentration (10 − 0.0078 mg/mL) for each available compound was built based on the UV signal: oxalic acid (*y* = 45.973 + 9 × 10 *x*; *R*^2^ = 0.9901); quinic acid (*y* = 46.061 + 610607 *x*; *R*^2^ = 0.9995); malic acid (*y* = 92.665 + 912,441 *x*; *R*^2^ = 0.999); citric acid (*y* = 45.682 + 1 × 10^6^ *x*, *R*^2^ = 0.9997), and succinic acid (*y* = 50.689 + 592888 *x*; *R*^2^ = 0.9996). The results were expressed in g per 100 g of fruits and stems dry weight.

#### 3.2.5. Tocopherols

For tocopherol determination, the methodology was applied accordingly with that previously described by the authors [36]. An HPLC system (Knauer, Smartline system 1000, Berlin, Germany) coupled to a fluorescence detector (FP-2020; Jasco, Easton, MD, USA) programmed for excitation at 290 nm and emission at 330 nm was used. Separation of the tocopherols isoform was achieved using a Polyamide II (250 mm·4.6 mmi.d.) normal-phase column from YMC Waters, operating at 30 °C. The mobile phase used was a mixture of *n-hexane* and ethyl acetate (70:30, *v/v*) at a flow rate of 1 mL/min. Tocol (Matreya, Pleasant Gap, State College, PA, USA) was used as internal standard, and the results were expressed in mg 100 g^−1^ dw.

#### 3.2.6. Carotenoids and Chlorophyll

A fine dried powder (150 mg) of the lyophilized material was energetically mixed with 10 mL of acetone–hexane solution (4:6) for 1 min and filtered through Whatman No. 4 filter paper. The absorbance of the filtrate was assessed spectrophotometrically (Agilent 8453 UV-visible spectrophotometer, Canada) using as preferred wavelengths 453, 505, 645, and 663 nm [38]. The content of β-carotene was calculated according to the following equation: β-carotene (mg/100 mL) = 0.216 × A_663_ − 1.220 × A_645_ − 0.304 × A_505_ + 0.452 × A_453_; lycopene (mg/100 mL) = −0.0458 × A_663_ + 0.204 × A_645_ − 0.304 × A_505_ + 0.452 × A_453_; Chlorophyll a (mg/100 mL) = 0.999 × A_663_ − 0.0989 × A_645_; Chlorophyll b (mg/100 mL) = −0.328 × A_663_ + 1.77 × A_645_, and further expressed in mg/100 g of fresh weight.

#### 3.2.7. Phenolic Compounds

The preparation of the hydroethanolic extracts (ethanol:water 80:20, *v/v*, 40 mL) was performed by two sequential macerations of the lyophilized okra pods sample (1 g), at 25 °C. The combined extracts were evaporated (Büchi R-210, Flawil, Switzerland) and further lyophilized. The phenolic compound chromatographic analysis was performed in a Dionex Ultimate 3000 UPLC (Thermo Scientific, San Jose, CA, USA) system equipped with a DAD (280 and 370 nm as preferred wavelength) detector, and coupled to a electrospray ionization mass detector (LC-DAD-ESI/MSn). The chromatographic separation of the compounds was achieved with a Waters Spherisorb S3 ODS-2 C18 (3 µm, 4.6 mm × 150 mm, Waters, Milford, MA, USA) column, operating at 35 °C. The elution solvents, working in gradient mode, were 0.1% formic acid in water and acetonitrile. Finally, for MS detection in negative mode, was used a Linear Ion Trap LTQ XL mass spectrometer (ThermoFinnigan, San Jose, CA, USA) equipped with an electrospray ionization (ESI) source. Nitrogen served as the sheath gas (50 psi); operating with a spray voltage of 5 kV, temperature of 325 °C, and a capillary voltage of −20 V [39]. The identification was completed using reported data from literature or by comparison with the available commercial standards (Extrasynthèse, Genay, France). Seven-level calibration curves for each available phenolic standard were constructed based on the UV signal for quantification analysis and the results expressed in mg per g of extract.

### 3.3. Bioactive Properties

#### 3.3.1. Antioxidant Potential

DPPH (2,2-diphenyl-1-picrylhydrazyl) free radical decolonization assay was performed using a UV–vis spectrophotometer (Perkin Elmer Lambda 40 UV/VIS Spectrophotometer), according to Barros et al. [37] and calculated as a percentage of discoloration using the formula: [(A_DPPH_ − A_S_)/A_DPPH_]/100, where A_DPPH_ is the absorbance of DPPH solution and A_S_ in the absorbance at 515 nm of the test sample. FRAP radical scavenging ability assay was realized via the method reported by Garcia et al. [21]. The absorbance was read at 734 nm and the scavenging capacity was calculated as a percentage of inhibition, according to the formula: [1 − (A_S_/A_control_)] × 100. Inhibition of β-carotene bleaching was evaluated through the β-carotene/linoleate assay according to Chahdoura et al. [40]; the neutralization of linoleate free radicals avoids β-carotene bleaching, which is measured using the formula: (β-carotene absorbance after 2 h of assay/initial absorbance) × 100.

#### 3.3.2. Cytotoxic Activity

Stock solutions of the extracts at 8 mg/mL were prepared in water and successive solutions were made from 0.005 to 0.4 mg/mL. The cytotoxic potential was evaluated in four human tumour cell lines: HeLa (cervical carcinoma), HepG2 (hepatocellular carcinoma), MCF-7 (breast adenocarcinoma), and NCI-H460 (non-small cell lung carcinoma). The cell lines were plated in 96-well plates, with a final density of 1.0 × 10^4^ cells/well, and were allowed to attach for 24 h. Next, different extract concentrations were added to the cells, which were incubated for 48 h. Both cells treatment and the Sulforhodamine B assay were carried out according to protocol established by Abreu et al. [41]. For the toxicity assessment toward liver cells, a primary cell culture (PLP2) was obtained from a freshly harvested porcine liver. Ellipticine (Sigma-Aldrich, St. Louis, MO, USA) was applied as positive control. For ethical control, the pig was slaughtered for human consumption and not for scientific purposes, and for that no Ethical Committee was required for the approval of this type of tissue manipulation.

#### 3.3.3. α-Amylase Inhibitory Assay

The α-amylase inhibition assay was conducted as described by Makino et al. [42] with slight modifications. In brief, the assay mixture constituted of 500 µL of 1% starch solution, 400 µL of 0.1 M sodium phosphate buffer (pH 7.0), 50 µL of okra pods dissolved in dimethyl sulfoxide (DMSO), and 50 µL of pancreatic a-amylase (Sigma, St. Louis, MO, USA) solution (2 U/mL). The mixture was incubated for 10 min at 37 °C. Thereafter, 3 mL of 3.5-dinitrosalicylic acid (DNS) color reagent were incorporated. The solution was then placed, for 5 min, in a boiling water bath and diluted with 20 mL of distilled water. The absorbance was recorded at 540 nm. The okra pods extract was tested for a-amylase inhibitory potential at different concentrations (10.0–0.15 mg/mL). A negative control sample was prepared as described, without adding the plant extract, while acarbose was used as a positive control. All data were expressed as percentage of inhibition using the following formula: ((A_control_ − A_sample_)/A_control)_ × 100, where A_control_ and A_sample_ were the absorbance values of the negative control and sample, respectively.

#### 3.3.4. α-Glucosidase Inhibitory Assay

The α-glucosidase inhibition assay was performed according to Cásedas, Les, Gómez-Serranillos, Smith, and López [43] with alterations. The α-glucosidase reaction mixture consisting of 2.5 mM 4-*p*-nitrophenyl-α-d-glucopyranoside (4-*p*NPG), 250 µL of extract at various concentrations in DMSO, and 0.3 U/mL of α-glucosidase in phosphate buffer (pH 6.9), was placed on a water bath at 37 °C for 15 min. DMSO, enzyme, and substrate served as negative controls, whereas, acarbose replaced the plant extract and was considered as a positive control. Absorbance of the resulting *p*-nitrophenol (pNP) was read at 405 nm and was considered representative of the enzyme activity. Okra extract was tested against α-glucosidase enzyme at distinct concentrations (5.0–0.15 mg/mL). The percentages of inhibition of okra extract and acarbose (I%) were expressed using the following formula: ((A_control_ − A_sample_)/A_control)_ × 100, where A_control_ and A_sample_ were the absorbance values of the negative control and the sample, respectively.

#### 3.3.5. Analgesic Activity

The method of Koster, Anderson, and De Beer [44] was adopted to assess the antinociceptive activity via the acetic acid abdominal constriction test (writhing test). *Swiss albino* mice were selected and divided into groups of six animals each. The control group was pretreated subcutaneously with 10 mL/kg of saline. The other group was pretreated, by the same route, with the reference drug acetyl salicylate of lysine (ASL) at 200 mg/kg. To the remaining groups, 10 mL/kg of acetic acid solution (1%) were injected intraperitoneally, 30 min after the administration of ethanol:water okra extract (80:20, *w*/*w*) at the doses of 10, 50, and 100 mg/kg. After acetic acid administration, the writhe number was enumerated for 30 min. Analgesic activity was expressed as inhibition percent of the usual number of writhes of the control animals. The percentages of inhibition were measured using the formula below: % inhibition = ((number of writhes)_control_ − (number of writhes)_treated group_) × 100)/(number of writhes)_control_. For ethical control, the animals were handled according to the guidelines of the Tunisian Society for the Care and Use of Laboratory Animals, and the study was approved by the University of Monastir Ethical Committee (Approval No: CER-SVS 007/2020 ISBM).

### 3.4. Statistical Analysis

The data of all the assays performed were expressed as mean values and standard deviations (SD), as a result of the three repetitions of the samples and concentrations. Significant differences were determined with α = 0.05 between the number of writhes and the controls used for the study of analgesic activity, applying the student’s t-test. Analyses were performed with the IBM SPSS Statistics for Windows, version 23.0. (IBM Corp., Armonk, New York, NY, USA).

## 4. Conclusions

To the best of the authors’ knowledge, this a pioneering report on the nutritional value, cytotoxic, antidiabetic, and analgesic potentials of Tunisian *A. esculentus*, and the first in-depth study on its phenolic and volatile profiles. The information herein reported not only corroborates the importance of the production and consumption in a daily-basis diet of this nutritious vegetable by the Tunisian population, but also suggests its exploitation as a source of high-added value molecules with antioxidant, cytotoxic, antidiabetic, and analgesic capacity that can be exploited by the pharmaceutical and food industries, with externalities for other industrial sectors. 

## Figures and Tables

**Table 1:** Proximate composition (g 100^−1^ g fw) and mineral content (mg 100^−1^ g fw) of the studied okra pods (mean ± SD).

Proximate composition (g 100^−1^ g fw)	Okra pods
Moisture	81.9 ± 0.1
Total ash	1.46 ± 0.04
Crude proteins	3.55 ± 0.03
Total fat	0.066 ± 0.001
Total available carbohydrate	4.864 ± 0.003
Total dietary fiber	8.2 ± 0.4
Insoluble dietary fiber	4.7 ± 0.4
Soluble dietary fiber	3.43 ± 0.07
Energy (kcal/100 g fw)	50.57 ± 0.02
**Mineral content (mg 100^−1^ g fw)**	
Fe^2+^	1.6 ± 0.2
Cu^2+^	0.49 ± 0.05
Mn^2+^	0.13 ± 0.01
Zn^2+^	2.0 ± 0.2
Mg^2+^	102 ± 4
Ca^+^	266 ± 20
Na^+^	127 ± 6
K^+^	337 ± 32

**Table 2:** Composition in sugars, organic acids (g 100 g^−1^ fw), tocopherols, and pigments (mg 100 g^−1^ fw) of the studied okra pods (mean ± SD).

Soluble Sugars		Organic Acid	
Fructose	34.8 ± 0.01	Oxalic acid	23.24 ± 0.01
Glucose	30.91 ± 0.01	Malic acid	20.92 ± 0.01
Sucrose	110.40 ± 0.02	Shikimic acid	tr
Trehalose	15.46 ± 0.02	Citric acid	54.64 ± 0.01
Total sugars	190.9 ± 0.05	Fumaric acid	3.53 ± 0.001
**Carotenoids**		Total organic acid	102.39 ± 0.01
β-carotene	0.83 ± 0.01	**Tocopherols**	
Lycopene	0.55 ± 0.01	α-Tocopherol	1.66 ± 0.01
**Chlorophylls**		γ-Tocopherol	2.76 ± 0.01
Chlorophyll a	3.53 ± 0.02	Total Tocopherols	3.97 ± 0.02
Chlorophyll b	2.43 ± 0.01		
Total chlorophyll	6.02 ± 0.03		

**Table 3:** Volatile compounds (%) identified in okra pods.

Compounds	L.R.I	Okra Pods
4-Hydroxy-d-methyl-2-pentanone	837	3.50 ± 0.10
α-Pinene	941	1.90 ± 0.05
(E)-2-Heptanal	962	0.70 ± 0.02
Benzaldehyde	963	0.50 ± 0.01
1-Octn-3-ol	981	3.10 ± 0.60
*β*-Pinene	982	0.90 ± 0.01
6-Methyl-5-hepten-2-one	987	2.40 ± 0.40
Limonene	1032	6.70 ± 0.30
(E)-3-Octen-1-ol	1062	4.10 ± 0.10
(E)-2-Octen-1-ol	1070	4.30 ± 0.10
1-Octanol	1075	4.00 ± 0.10
*n*-Undecane	1100	0.70 ± 0.01
Linalool	1101	1.60 ± 0.07
Nonanal	1104	3.40 ± 0.10
*cis*-ρ-Menth-2-en-1-ol	1123	1.60 ± 0.04
Camphor	1145	3.40 ± 0.10
Menthol	1174	1.50 ± 0.05
α-Terpineol	1191	0.90 ± 0.03
*cis*-Dihydrocarvone	1195	0.60 ± 0.01
*n*-Dodecane	1200	2.60 ± 0.03
Decanal	1206	4.60 ± 0.20
*β*-Cyclocitral	1222	1.80 ± 0.04
Exo-fenchyl acetate	1230	2.20 ± 0.06
Carvone	1244	4.50 ± 0.10
(E)-Anethole	1285	6.90 ± 0.30
*n*-Tridecane	1300	2.00 ± 0.03
α-Cubebene	1351	1.70 ± 0.05
Eugenol	1358	4.20 ± 0.20
*n*-Tetradecane	1400	2.10 ± 0.04
*β*-Caryophyllene	1419	5.40 ± 0.20
2,5-Dimethoxy-ρ-cymene	1424	1.60 ± 0.04
Δ8,9-Dehydro-4-hydroxythymol dimethyl ether	1444	4.30 ± 0.20
Alloaromadendrene	1462	2.60 ± 0.05
γ-Muurolene	1478	1.70 ± 0.08
3,4-Dehydro-β-ionone	1786	2.50 ± 0.10
Monoterpene hydrocarbons		9.50 ± 0.16
Oxygenated monoterpenes		22.20 ± 0.05
Sesquiterpene hydrocarbons		11.40 ± 0.07
Phenylpropanoids		11.10 ± 0.12
Apocarotenes		4.30 ± 0.04
Non-terpene derivatives		38.00 ± 0.12
Total identified (%)		96.50 ± 0.02

LRI: linear retention indices on DB-5 column. Data expressed as means ± standard deviations.

**Table 4:** Retention time (Rt), wavelengths of maximum absorption in the visible region (λmax), mass spectral data and tentative identification, and phenolic compounds quantification (mg g of extract) in okra pods.

Peak	Rt (min)	λmax (nm)	Molecular Ion[M − H]^−^ (*m/z*)	MS^2^ (*m/z*)	Tentative Identification	Quantification
1	14.84	354	625	301(100)	Quercetin-dihexoside	0.785 ± 0.001
2	15.75	353	595	301(100)	Quercetin-*O*-pentosyl-hexoside	0.90 ± 0.020
3	18.67	353	463	301(100)	Quercetin-3-*O*-glucoside	1.500 ± 0.010
4	19.69	352	505	463(23), 301(100)	Quercetin-*O*-acetylhexoside	0.628 ± 0.001
5	20.27	350	505	463(35), 301(100)	Quercetin-*O*-acetylhexoside	0.175 ± 0.003
6	22.13	340	447	285(100)	Kaempferol-3-*O*-glucoside	0.139 ± 0.004
7	32.81	314	593	285(100)	Kaempferol-*O*-caffeoyl-deoxyhexoside	0.463 ± 0.002
8	37.98	314	635	489(21), 285(100)	Kaempferol-*O*-deoxyhexoside-*O*-acetyl-caffeoyl	0.148 ± 0.001
					Total phenolic compounds	4.740 ± 0.030

Standard calibration curve used for all the flavonols: quercetin 3-*O*-glucoside (*y* = 34843 *x* − 160173; *R*^2^ = 0.9998; LOD = 0.21 μg/mL; LOQ = 0.71 μg/mL). Linearity was performed with 11 levels calibration curve (0.1–100 μg/mL).

**Table 5:** Bioactivity of Tunisian okra pods (*Abelmochus esculentus* L. Moench).

		*Abelmoschus esculentus* L. Moench	
Antioxidant activity; EC_50_ = mg/mL ^A^			
DPPH scavenging activity		1.03 ± 0.02	
FRAP scavenging activity		0.89 ± 0.03	
β-carotene bleaching inhibition		0.47 ± 0.02	
Cytotoxic activity; IC_50 =_ mg/mL ^B^			
NCl-H460		49.62 ± 0.02	
MCF-7		56.40 ± 0.30	
HeLa		67.30 ± 0.50	
HepG-2		168.00 ± 7.00	
Anti-hyperglycaemic activity; IC_50 =_ mg/mL ^C^			
α-amylase inhibition		0.125 ± 0.02	
α-glucosidase inhibition		0.110 ± 0.01	
**Analgesic Activity**			
**Groups**	**Concentration (mg/kg)**	**Number of Writhes**	**Inhibition of Writhing (%)**
Control	-----	81.20 ± 0.40	------
Tunisian okra pods	10	59.70 ± 0.60 **	26.48
	50	47.00 ± 0.40 **	42.09
	100	34.00 ± 0.30 ***	58.11
Reference drug (ASL)	200	30.00 ± 1.00 ***	63.65

Values are expressed as mean ± S.E.M. (*n* = 6); ASL: Acetylsalicylate of lysine. ^A^ Trolox EC_50_ values: 0.030 ± 0.001 mg/mL (DDPH), 0.072 ± 0.002 mg/mL (FRAP), and 0.010 ± 0.001 mg/mL (β-carotene bleaching inhibition); ^B^ Ellipticine GI_50_ value: 0.91 ± 0.04 μg/mL (MCF-7), 1.03 ± 0.09 μg/mL (NCI-H460), 1.91 ± 0.06 μg/mL (HeLa), 1.1 ± 0.2 μg/mL (HepG2). ^C^ Acarbose IC_50_ values: 0.093 ± 0.001 mg/mL (α-amylase inhibition) and 0.28 ± 0.01 mg/mL (α-glucosidase inhibition). ** *p* ≤ 0.01 significant from control; *** *p* ≤ 0.001 significant from control.
